# Use of Droplet Digital Polymerase Chain Reaction to Identify Biomarkers for Differentiation of Benign and Malignant Renal Masses

**DOI:** 10.3390/cancers16040787

**Published:** 2024-02-15

**Authors:** Joshua P. Hayden, Adam Wiggins, Travis Sullivan, Thomas Kalantzakos, Kailey Hooper, Alireza Moinzadeh, Kimberly Rieger-Christ

**Affiliations:** 1 Department of Urology, Lahey Hospital & Medical Center, Burlington, MA 01805, USA; joshua.p.hayden@lahey.org (J.P.H.); adam.b.wiggins@lahey.org (A.W.); alireza.moinzadeh@lahey.org (A.M.); 2 Department of Translational Research, Lahey Hospital & Medical Center, Burlington, MA 01805, USA; travis.b.sullivan@lahey.org (T.S.); kalantzakos@livemail.uthscsa.edu (T.K.); kailey.e.hooper@lahey.org (K.H.)

**Keywords:** microRNA, ddPCR, renal cell carcinoma, cell-free biomarker, benign

## Abstract

**Simple Summary:**

Differentiating between benign and malignant renal neoplasms is critical to clinical management and can significantly impact treatment. Most contemporary methods involve invasive tissue biopsy of the renal mass. One potential less invasive method for discriminating between various histologic types involves the use of cell-free biomarkers such as microRNA (miRNA). Several studies using conventional real-time PCR (RT-PCR) analysis have identified various blood-based miRNA biomarkers of renal cell carcinoma (RCC). However, few of these studies report biomarkers distinguishing benign masses. We utilized droplet digital PCR (ddPCR), a robust nucleic acid quantification method, to examine the potential use of plasma miRNA as biomarkers of renal tumors. We demonstrate that the combination of miR-210-3p and miR-222-3p is higher in the blood of patients with RCC than in benign patients. In addition, this study demonstrated that ddPCR outperformed conventional RT-PCR in detecting miRNAs that were characteristic of malignant versus benign renal masses.

**Abstract:**

Several microRNAs (miRNAs) have been identified as cell-free biomarkers for detecting renal cell carcinoma (RCC). Droplet digital polymerase chain reaction (ddPCR) is a unique technology for nucleic acid quantification. It has the potential for superior precision, reproducibility, and diagnostic performance in identifying circulating miRNA biomarkers compared to conventional quantitative real-time PCR (qRT-PCR). This study aims to evaluate the performance of ddPCR compared to qRT- PCR in identifying miRNA biomarkers that differentiate malignant from benign renal masses. Potential biomarkers of RCC were identified from a literature review. RNA was extracted from the plasma of 56 patients. All the samples underwent analysis via ddPCR as well as qRT-PCR, and expression levels were recorded for the following miRNAs: miR-93, -144, -210, -221, and -222. Tumors were grouped into low-grade ccRCC, high-grade ccRCC, papillary RCC, and benign masses (primarily angiomyolipoma). The miRNA miR-210 (*p* = 0.034) and the combination of miRs-210 and miR-222 (*p* = 0.003) were expressed at significantly higher rates among those with RCC than those with benign masses, as measured by ddPCR. Using the combination of miR-210 and miR-222, ddPCR identified significant differences between the subgroups: papillary RCC versus benign (*p* = 0.03), low-grade ccRCC versus benign (*p* = 0.026), and high-grade ccRCC versus benign (*p* = 0.002). The only significant difference between these subgroups using qRT-PCR was between high-grade ccRCC and benign (*p* = 0.045). All the AUCs were significant when comparing each RCC subgroup with benign for both PCR technologies. Using a combination of miR-210 and miR-222, ddPCR identified significant differences between benign and malignant renal masses that were not identified as significant by conventional qRT-PCR.

## 1. Introduction

Renal cell carcinoma (RCC) is the ninth most common cancer diagnosed worldwide, and its detection has increased with the growing use of abdominal computerized tomography (CT) for diagnostic and screening purposes [1]; clear cell (ccRCC) and papillary (pRCC) are the most common histological subtypes. Other benign masses can also arise from the renal parenchyma that can have similar radiographic findings to RCC. Among these benign masses are angiomyolipoma (AML), a type of benign renal mass that may be indolent or may grow to cause mass effect or spontaneously hemorrhage, and oncocytoma, a mass with low malignant potential that can be difficult to discern from chromophobe RCCs [1]. As such, differentiation between benign and malignant renal masses is a sometimes challenging but nonetheless important step in managing renal masses, as this impacts prognosis and treatment. In summary, renal masses exist on a continuum, most of them have their own distinct subtypes, and all have distinct malignant potential and potential for harm to the patient.

While there are multiple ways to differentiate between benign and malignant renal masses, whether it be radiographically or with renal mass biopsy, these are imperfect strategies with variable sensitivity and specificity for detecting RCC and can be invasive and in rare cases cause harm to the patient [2]. Therefore, the ideal test for differentiating between benign and malignant renal masses would be one with high sensitivity and specificity that is minimally invasive and readily available. To this end, a blood-based test that has the capacity to differentiate benign from malignant renal masses would have the potential for a profound impact on RCC management.

One example of blood-based tests that could aid in diagnosing and managing urologic cancers is a test that enables the identification of microRNAs (miRNAs). These small non-coding RNA molecules exert epigenetic control on the mRNAs transcribed from genes implicated in the pathogenesis of various urologic malignancies. In testicular cancer, for example, the microRNA miR-371a-3p has shown promise as a serum-based biomarker that has improved sensitivity and specificity compared to other serum tumor markers for the detection of macroscopic testicular germ cell tumors of distinct histologies, except for teratoma [3]. With respect to RCC, studies have identified dysregulated miRNAs in various types of kidney masses and predicted which malignant tumors progress to metastatic disease [4,5]. In addition, several miRNAs have been identified as cell-free biomarkers for detecting RCC [6,7,8].

Droplet digital polymerase chain reaction (ddPCR) is a novel nucleic acid quantification technology. It has the potential for superior precision, reproducibility, and diagnostic performance in identifying circulating miRNA biomarkers for cancer compared to conventional quantitative real-time PCR (qRT-PCR) [9]. This study aims to evaluate the performance of ddPCR in identifying miRNA biomarkers that differentiate malignant from benign renal masses and compare its performance to conventional qRT-PCR. The ability to distinguish between malignant and benign renal masses using a minimally invasive, blood-based test could ultimately reduce the morbidity associated with renal mass biopsy, which is imperfect in identifying malignant versus benign renal masses. Furthermore, the successful development of a blood-based biomarker holds the promise of further decreasing morbidity by reducing surgeries that may be unwarranted.

## 2. Materials and Methods

### 2.1. Study Design

This study was performed according to the guidelines and regulations of the Institutional Review Board (IRB) protocol 2011-048 at the Lahey Clinic, Inc., Burlington, MA, USA, a tertiary medical facility. All patients presenting to our Urology Department for treatment of a renal mass between 2014–2021 were approached for participation in this study. All patients provided signed consent for using their blood for research purposes. We prospectively collected blood samples from patients. Inclusion criteria for this study were patients greater than 18 years of age undergoing an extirpative surgical procedure such as nephrectomy or partial nephrectomy for management of a renal neoplasm. Exclusion criteria were prior or concomitant non-renal malignancy and known genetic predisposition to renal or other malignancy (e.g., Lynch syndrome, von Hippel-Lindau syndrome).

Candidate biomarkers of renal cell carcinoma were identified through an extensive literature review of renal cell carcinoma cell-free biomarkers in serum or plasma via PubMed of publications up to December of 2021, and a consensus list of biomarkers was compiled for testing via ddPCR.

### 2.2. Plasma Samples

Blood samples were collected from patients in K2 EDTA vacutainers (Becton Dickinson, Franklin Lakes, NJ, USA) at the time of surgery. Cell-free plasma samples were prepared using a double centrifugation protocol to minimize the presence of cellular debris. The blood samples were first centrifuged at 1870× *g* for 10 min at 4 °C without breaking. The resulting supernatant was then transferred to a 15 mL conical and centrifuged at 1870× *g* for 15 min at 4 °C without breaking. The resulting double-spun plasma was then aliquoted into tubes for storage at −80 °C.

The occurrence of hemolysis during blood collection and plasma processing can lead to increased plasma levels of red blood cell-derived miRNAs that can significantly affect the measurement and normalization of cell-free miRNA [10]. Therefore, all plasma samples were measured via spectrophotometry at 415 nm to evaluate the level of hemolysis, and samples with an absorbance > 1.2 were excluded from the analysis [11].

### 2.3. RNA Isolation

Total RNA was isolated from 200 μL of plasma using the miRNeasy Serum/Plasma Advanced Kit (Qiagen, Hilden, Germany), following the manufacturer’s recommended protocol with the following modifications. The lysis reagent RPL was supplemented with 1 μg of MS2 phage RNA (Roche, Basel, Switzerland) as carrier and 1 μL of synthetic cel-miR-39 (Norgen, Thorold, ON, Canada) to serve as a spike-in control to monitor isolation efficiency. The purified RNA was eluted in 23 μL of nuclease-free water and stored at −80 °C.

### 2.4. PCR Analyses

#### 2.4.1. cDNA Synthesis

Reverse transcription (RT) for miRNA analysis was performed using miRCURY LNA RT Kit (Qiagen, Hilden, Germany) and TaqMan™ MicroRNA RT Kit (Applied Biosystems, Foster City, CA, USA) according to the manufacturer’s protocols with the following modifications. Reverse transcription reactions utilized 4 μL of RNA in a 20 μL reaction for miRCURY and 5 μL of RNA in a 15 μL reaction for TaqMan.

#### 2.4.2. ddPCR

The QX200 Droplet Digital PCR System with manual droplet generation and QX Manager software (Version 1.2, Bio-Rad) was used for ddPCR analysis. Both miRCURY LNA assays and TaqMan assays were tested to evaluate their performance in ddPCR. For the miRCURY LNA assays, each 24 μL ddPCR reaction was composed of 12 μL of 2× EvaGreen Supermix (Bio-Rad, Hercules, CA, USA) and 1.2 μL of assay. For the TaqMan assays, each 24 μL ddPCR reaction was composed of 12 μL of 2× Supermix for probes (Bio-Rad) and 1.2 μL of assay. Each reaction was vortexed, and 20 μL was loaded into a droplet manifold along with 70 μL of the appropriate droplet oil and subsequently loaded into the droplet generator. Each ddPCR assay was optimized by performing titrations of the cDNA input, as well as annealing/extension temperature gradient testing.

After droplet generation, 40 μL of the resulting droplets were transferred into a 96 deep-well PCR plate (Bio-Rad), sealed with a foil seal, and the PCR was performed on a deep-well C1000 thermal cycler (Bio-Rad), as follows. Conditions for the miRCURY LNA assays were 95 °C for 5 min, 40 cycles of 95 °C for 30 s and annealing/extension for 1 min, then 95 °C for 30 s, 4 °C for 5 min, 90 °C for 5 min, and back to 4 °C: ramp 1.6 °C/s. Thermal cycling conditions for the TaqMan assays were 95 °C for 5 min, 40 cycles of 95 °C for 15 s and annealing/extension for 1 min, then 95 °C for 15 s, 4 °C for 5 min, 90 °C for 5 min, and back to 4 °C: ramp 2 °C/s. Results were normalized to the geometric mean of control miRNAs [12].

#### 2.4.3. Conventional Real-Time PCR (RT-PCR)

RT-PCR analysis was performed in 20 μL reactions using the miRCURY LNA miRNA PCR Assays (Qiagen) and TaqMan™ MicroRNA Assays (ABI, Prospect, Australia) according to the manufacturer’s protocols with the following modification. An aliquot of the miRCURY RT was diluted 1:10 prior to use in RT-PCR. Thermal cycling reactions were performed on the CFX96 Touch Real-Time System (Bio-Rad). Results were normalized to the average of control miRNAs and quantified as x = 2^−ΔCt^.

#### 2.4.4. Selection of Normalizers

Endogenous control miRNAs serving as normalizers were included for use in data analysis. We sought to identify miRNA with robust and uniform expression across all the samples in this study. A list of candidate miRNA normalizers was identified for testing from a previous study that surveyed three serum-based analyses, each including over 50 healthy control samples [13].

#### 2.4.5. Limit of Quantification

Serial dilution (1:2) of cDNA generated from plasma RNA was performed to determine the limit of quantification (LOQ) for the ddPCR assays. The LOQ was defined as the lowest concentration that could be detected with a CV ≤ 25% [14].

### 2.5. Statistical Analyses

Categorical data were compared using the Fisher’s Exact test. The continuous data were compared using the Mann–Whitney U test or the Kruskal–Wallis one-way analysis of variance, as appropriate. A *p*-value < 0.05 was considered statistically significant. All analyses were performed using SPSS v29 (IBM Corp., Armonk, NY, USA).

## 3. Results

### 3.1. Study Cohort

There were 67 samples that qualified for this study based on our clinical inclusion and exclusion criteria. Four groups of samples were identified: patients with a benign renal mass, those with pRCC, low-stage ccRCC (stages I–II), and high-stage ccRCC (stages III–IV). Of these, 3 were excluded due to a high level of hemolysis in the plasma sample, and 11 were excluded due to ddPCR values below the LOQ for several assays. Of the 53 samples remaining in the study (Table 1), 44 were diagnosed with RCC, and 9 were benign renal masses: 4 of these were biopsied prior to resection. Of the RCC cases, 17 were pRCC (15 with low-stage and 2 with high-stage), and 27 were ccRCC (14 with low-stage, and 13 with high-stage). The benign cases comprised AML (n = 7), along with one case of arteriovenous malformation and one of multilocular cystic neoplasm of low malignant potential. The RCC patients were significantly older than the benign patients, and the ccRCC tumors were significantly larger than the pRCC and benign masses. The variables of age, BMI, and sex were insignificant between the groups.

### 3.2. Candidate Biomarkers

We identified 35 previous studies of blood-based biomarkers of RCC. At least two of these studies identified 17 miRNAs as potential biomarkers (Supplementary Materials). The predominant biomarker identified was miR-210-3p, which was found to be elevated in RCC patients in seven of these studies. These prior analyses primarily compared miRNA biomarker levels in the blood of healthy controls and patients with RCC. In addition, one study compared healthy controls and patients with AML, and it identified miR-144-3p as a potential biomarker for these cohorts.

### 3.3. PCR

#### 3.3.1. Benign Versus RCC

**Figure 1:**
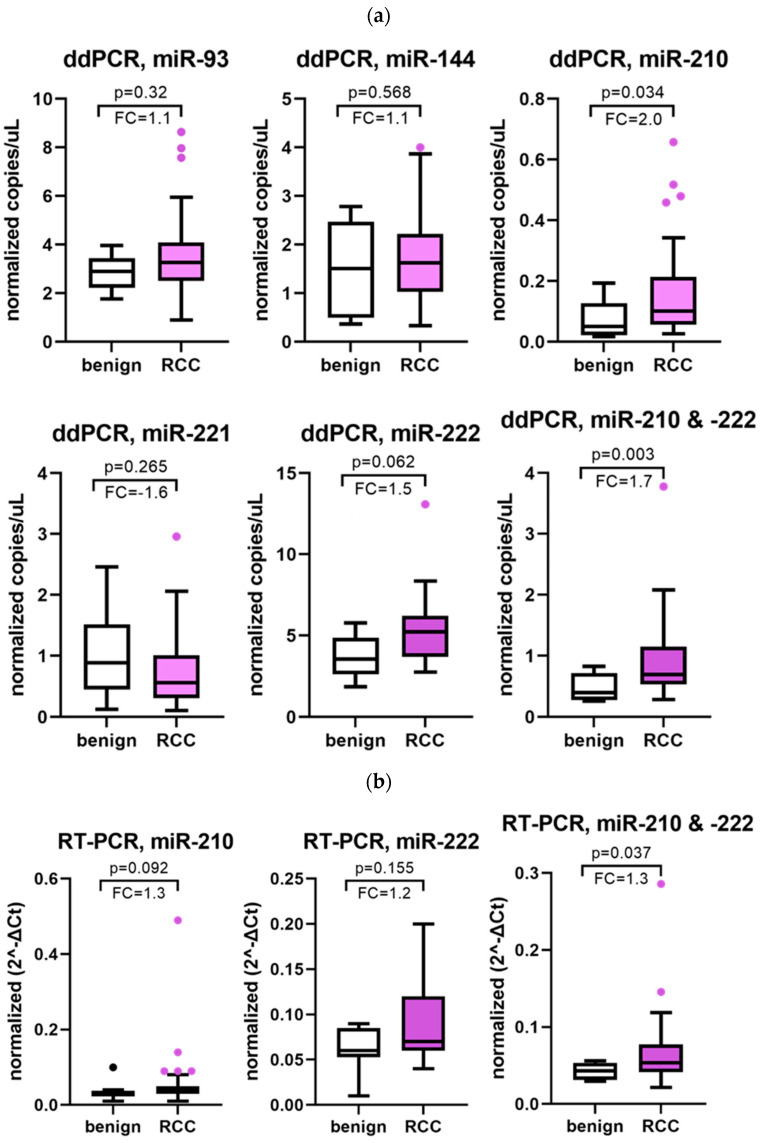

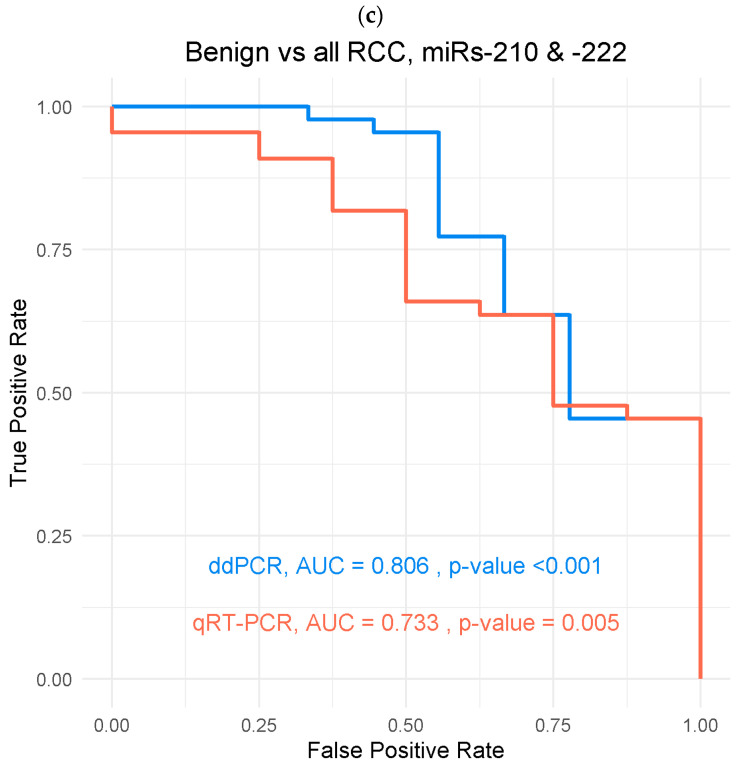
Expression levels of each of the miRNA in this study. The median fold change (FC) in expression for the RCC cohort compared to benign, as analyzed (**a**) by ddPCR and (**b**) by conventional RT-PCR. (**c**) ROC curves for the combination of miRs -210-3p and -222-3p as analyzed by ddPCR and conventional qRT-PCR, comparing benign and all RCC samples.

The reaction conditions of seven assays were optimized for ddPCR detection (Supplementary Materials), including two for use as normalizers (miRs 103-3p and miR-16-5p). Of all the candidate biomarkers tested (Figure 1), only the level of miR-210-3p was significantly different in the plasma of RCC patients compared to benign (*p* = 0.034). Combinations of the various assay results were evaluated, using the geometric means, and the combination of miRs-210-3p and -222-3p was also significantly elevated in the plasma of RCC patients compared with benign (*p* = 0.003), with an AUC of 0.806 (95%CI 0.647–0.964, *p* < 0.001).

The levels of miRs-210-3p and -222-3p were also evaluated using conventional RT-PCR with this sample set to compare platform performance. Neither miR was significantly elevated when using this technology when evaluated alone (Figure 1). However, the combination of miRs-210-3p and -222-3p was significantly higher in the RCC group versus benign (*p* = 0.037), with an AUC of 0.733 (95%CI 0.571–0.895, *p* = 0.005).

#### 3.3.2. Subgroup Comparisons

**Figure 2:**
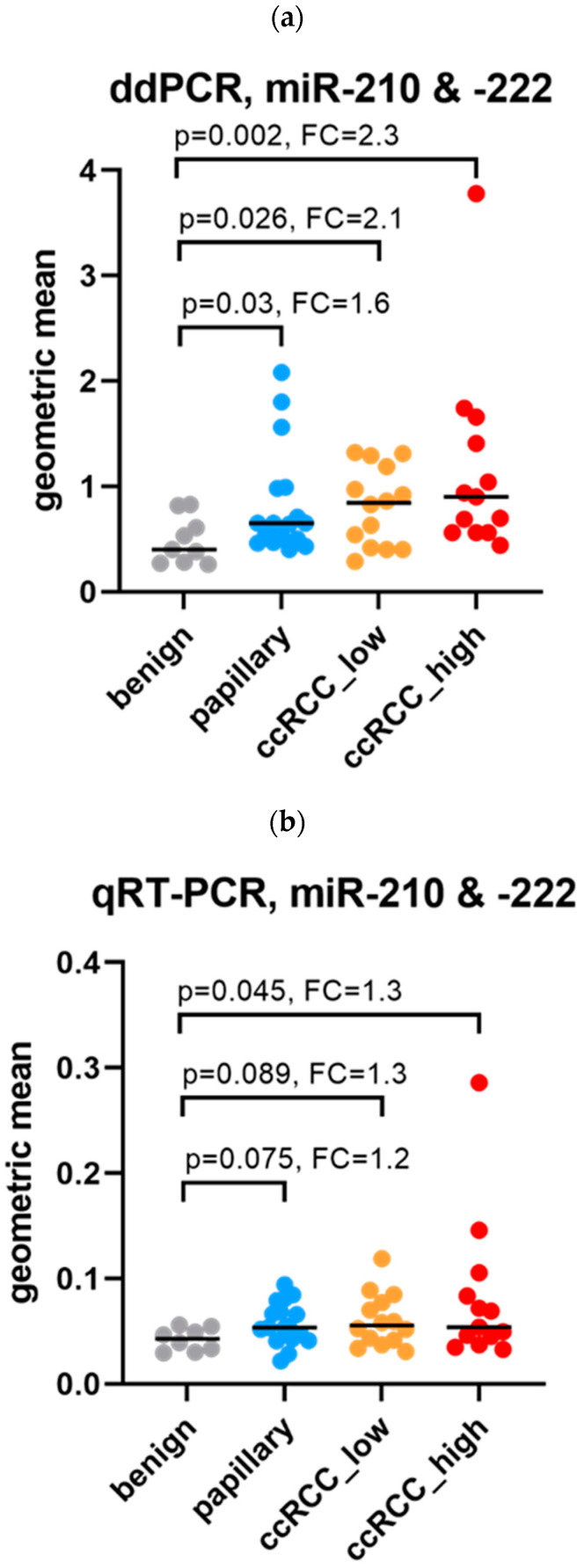

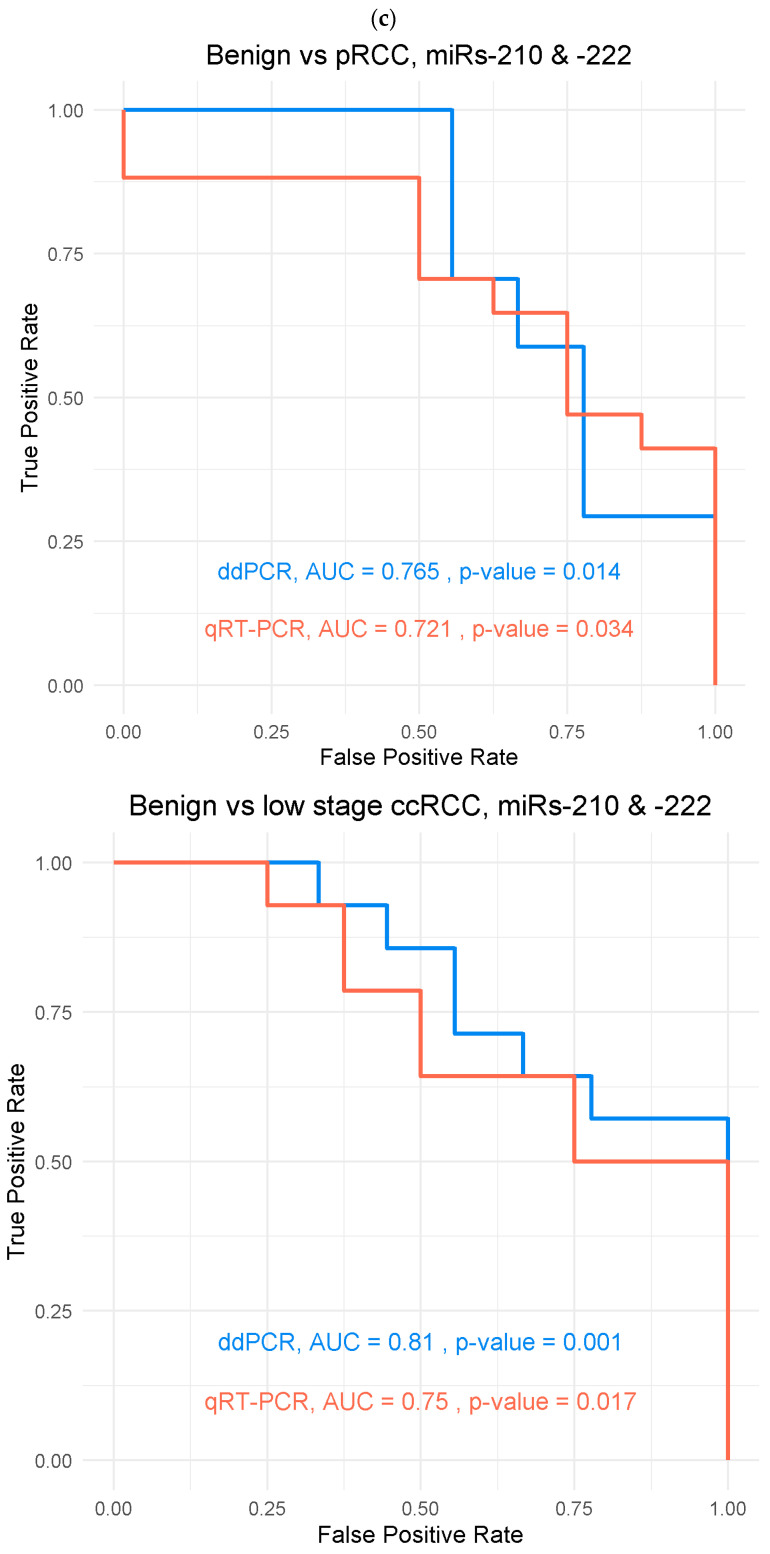

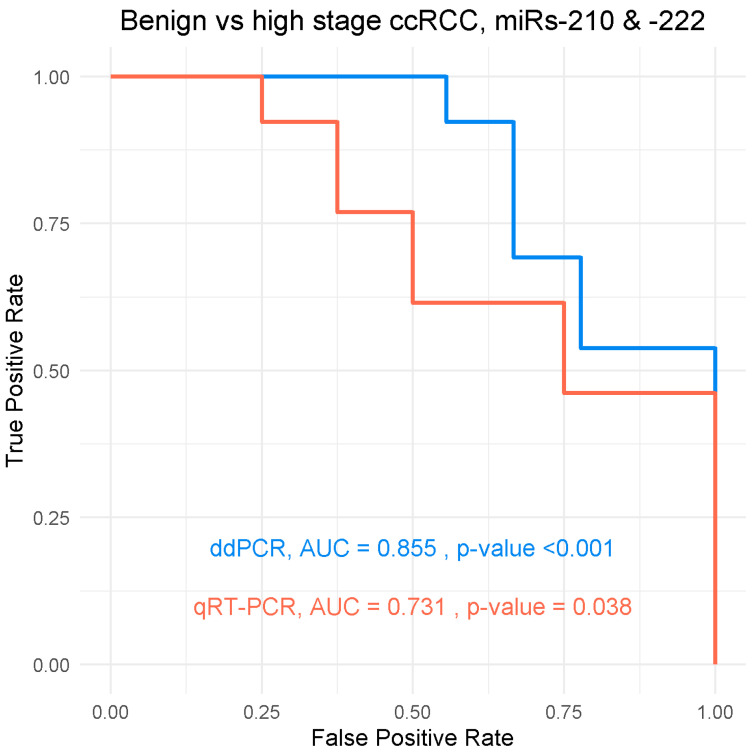
Subgroup comparisons using the combination of miRs -210-3p and -222-3p. The median fold change (FC) in expression for each of the RCC cohorts compared to benign, as analyzed by (**a**) ddPCR and (**b**) conventional RT-PCR. No significant differences were observed between RCC cohorts using either analysis platformed. (**c**) ROC curves for the combination of miRs -210-3p and -222-3p as analyzed by ddPCR and conventional qRT-PCR, comparing each of the RCC cohorts to benign.

Using ddPCR, comparisons between the subgroups revealed that the miR combination -210-3p and -222-3p was significantly elevated in all the RCC groups compared to benign (Figure 2): the levels were highest in high-stage ccRCC, followed by low-stage ccRCC, and then pRCC. The levels observed between the RCC groups were insignificant. ROC curve analyses resulted in significant AUCs for each RCC group compared with each benign.

For conventional RT-PCR, a similar trend to ddPCR was revealed: the levels were highest in ccRCC, followed by pRCC (Figure 2). However, the only significant difference observed was for high-stage ccRCC compared to benign. The levels observed between the RCC groups were insignificant. The ROC curve analyses were significant for each RCC group compared with each benign.

## 4. Discussion

Renal masses exist along a spectrum, including completely benign masses, masses with low malignant potential, and renal malignancies. Of course, the nature of the renal mass impacts prognosis, counseling, and management, including extirpative treatment and active surveillance. Data show that masses with benign histologies comprise 15–20% of renal masses smaller than 7 cm, with the remaining 80–85% containing malignant cells [15]. Benign renal masses can be subclassified based on the cell type of origin. Among the most common of these are AML and oncocytoma. Alternative diagnostic tools to discern between oncocytoma and other renal neoplasms, such as Sestamibi scans, have shown promise but have their own limitations, including exposing the patient to radiation [16]. AMLs, in contrast, are mesenchymal masses that form around small blood vessels and, as their name implies, are composed of vascular, smooth muscle, and fat cells. While classic triphasic AMLs contain macroscopic fat and are therefore readily distinguishable from RCC on CT, epithelioid AMLs lack macroscopic fat, are more difficult to differentiate from RCC on imaging, and harbor increased malignant potential, up to 33% in some series [17]. Moreover, distinguishing among renal masses is not always feasible using imaging techniques or even microscopically after renal mass biopsy, which can be nondiagnostic in approximately 15% of cases [18]. Therefore, identifying distinct miRNA expression patterns in the blood that can aid in the differentiation of benign and malignant renal masses would contribute to improved diagnostic accuracy and potentially guide appropriate therapeutic interventions.

The identification of reliable blood-based biomarkers has proven to be a critical tool for the early detection and prognosis of various cancers, including RCC [7,19,20,21]. In this study, we explored the potential role of miRNAs as plasma biomarkers to differentiate between benign and malignant renal masses, including RCC. Our results suggest that certain miRNAs are differentially expressed in the plasma of patients with benign versus malignant renal masses. Specifically, miR-210-3p (*p* = 0.034) and the combination of miR-210-3p and miR-222-3p (*p* = 0.003) were expressed at significantly higher rates among those with RCC compared to those with benign masses, primarily AML, as measured by ddPCR.

Several miRNAs have been identified as potential biomarkers for kidney tumors in various sample types (e.g., blood, tissue, and urine) [22]. With respect to blood-based biomarkers, these studies primarily emphasize differentiating RCC from healthy controls, while the focus of our study was to examine the utility of circulating biomarkers to distinguish malignant and benign renal masses. This distinction could serve as a valuable aid in the urologic setting to help guide treatment. Of the various miRNAs that have been reported as cell-free biomarkers of RCC, miR-210-3p is the best documented. It has been reported to be elevated in the serum of RCC patients in several studies [21,22,23,24,25], including serum exosomes [26] and urine [6]. Likewise, the level of miR-222-3p was found to be elevated in the blood of RCC patients [27], and its circulating levels were associated with a significantly reduced time-to-progression and a higher risk of an early relapse [28]. In addition, miR-222-3p was identified in a study of tumor tissue by Youssef et al. [29] as part of a classification system differentiating various RCC subtypes.

To potentially enhance the ability to use miRNA as biomarkers, we employed ddPCR as a relatively novel molecular technique for identifying blood-based biomarkers associated with benign and malignant renal masses. The use of ddPCR offers advantages over traditional qRT-PCR, such as increased precision and absolute quantification, making it an ideal method for detecting rare and low-abundance biomolecules in complex biological samples that may be limited in quality, such as serum or plasma [9]. In our study, ddPCR demonstrated superiority over qRT-PCR in identifying miRNAs distinctly expressed in malignant versus benign renal masses. This was especially apparent upon subgroup analysis, where ddPCR demonstrated superior detection of miRs that distinguish benign from each RCC type examined in this study. The ability of ddPCR to distinguish benign samples from two common malignant sub-types of RCC (papillary and clear cell), as well as early- and late-stage cancer, demonstrates that ddPCR is a robust means of biomarker analysis, and it may be useful in the clinical management of RCC. To our knowledge, this is the first study to utilize ddPCR to differentiate between benign and malignant renal masses. Our study highlights the enhanced performance of ddPCR, particularly in the context of low-abundance miRNAs, over traditional qRT-PCR methods in the identification of miRNA biomarkers for renal neoplasms, and it underscores the importance of continuing to adopt advanced molecular techniques for biomarker discovery, to ultimately provide diagnostic and treatment benefit for patients with renal neoplasms. Future work should expand among known miRNA biomarkers for RCC and other renal masses, utilizing ddPCR technology to identify individual miRNAs or miRNA combinations that could predict a renal mass’s malignant potential.

The strengths of the present study include the use of ddPCR technology, which has enhanced the ability of miRNA detection compared to previous work that has primarily identified miRNA biomarkers for RCC utilizing qRT-PCR, as well as our focus on differentiating benign masses. A unique aspect of this study is that it utilized double-spun plasma samples and validated biomarkers identified in prior studies of serum samples. Moreover, our cohort comprised patients with different subtypes of benign renal masses and subtypes of RCC. Limitations of our study include its single-institution, retrospective nature and the size of our cohorts. Future work could combine prospective samples collected across multiple institutions for improved detection of distinct classes of renal masses, as well as samples collected pre- and post-resection.

## 5. Conclusions

In conclusion, the application of relatively novel, advanced molecular techniques like ddPCR provides an avenue for improved identification and validation of cell-free biomarkers for differentiation among renal masses. This, in turn, could have significant clinical implications for the management of patients with either benign or malignant renal neoplasms.

## Figures and Tables

**Table 1:** Clinical and pathological characteristics for all of the samples included in this study.

Clinicopathologic Findings	Clear Cell n = 27	Papillary n = 17	Benign n = 9	*p* Value
Stage/type				
pT1-pT2	14	15	-	
pT3-pT4	13	2	-	
AML	-	-	7	
other	-	-	2	
Sex				0.743
Female	11 (41)	5 (29)	4 (44)	
Male	16 (59)	12 (71)	5 (56)	
Age at diagnosis	60 (53–68)	66 (57–73)	51 (42–59)	0.013
BMI at surgery	29.2 (25.2–34.3)	27.5 (24.2–32.3)	29.8 (24.7–32.3)	0.773
Tumor size, cm	6.3 (3.4–9.0)	3.1 (2.6–4.9)	2.6 (1.7–3.2)	0.009

## Data Availability

All data are available from the authors upon request.

## Supplementary Materials

The following supporting information can be downloaded at https://www.mdpi.com/article/10.3390/cancers16040787/s1, Table S1: Candidate Biomarkers, Table S2: ddPCR Reaction Details, Figure S1: Limit of Quantification. References [7,14,21,22,23,24,25,26,27,28,30,31,32,33,34,35,36,37,38,39,40,41,42,43,44,45,46,47,48,49,50,51,52,53,54,55] have been cited in the Supplementary Materials.
