# Supplementary material for: Use of Droplet Digital Polymerase Chain Reaction to Identify Biomarkers for Differentiation of Benign and Malignant Renal Masses
Source: Cancers (Basel). 2024 Feb 15;16(4):787. doi: 10.3390/cancers16040787 (PMC10886675; doi:10.3390/cancers16040787)
Supplement: Supplementary file 1 [file cancers-16-00787-s001.zip › cancers-2862989-supplementary.pdf]

Use of Droplet Digital Polymerase Chain Reaction to Identify Biomarkers for Differentiation of Benign and Malignant Renal Masses

Joshua P. Hayden[1], Adam Wiggins[1], Travis Sullivan[2], Thomas Kalantzakos[2], Kailey Hooper[2], Alireza Moinzadeh[1], Kimberly Rieger-Christ[1,2,*]

Supplemental Materials

Table S1. Candidate Biomarkers.
Candidate biomarkers of renal cell carcinoma were identified through extensive literature review of renal cell carcinoma cell free biomarkers in serum or plasma via PubMed of publications up to December of 2021. We identified 35 previous studies of blood-based biomarkers of RCC. Within these studies, 17 miRNA were identified as potential biomarkers in at least two of these studies. Supplemental Table 1: The biomarker studies surveyed to identify candidates for testing by ddPCR in this study.

	study	sample matrix	analysis platform	cohorts	candidate miRNA biomarkers
1	PMID: 33122915	plasma exosomal miRNA	NGS validated by qRT-PCR	RCC (pap and clear) vs control	upregulated: 1307-3p, 1469, 149-3p, 211-5p, 424-3p, 4279, 4501, 4653-3p, 4664-3p, 4707-5p, 4721, 5189-5p, 663a, 6718-5p, 6822-5p, 885-3p, 9901<br>downregulated: 21-5p, 29a-3p, 342-3p, 92a-1-5p
2	PMID: 27633984	plasma	microarray validated by qRT-PCR	ccRCC vs AML vs control	upregulated:144-3p<br>downregulated:
3	PMID: 33151036	plasma	microarray validated by qRT-PCR	up in ccRCC plasma	upregulated:483-5p<br>downregulated:
4	PMID: 31901870	plasma	qRT-PCR	ccRCC before and after resection	upregulated: miR-765<br>downregulated:
5	PMID: 23619562	plasma	qRT-PCR	down in RCC vs control	upregulated:<br>downregulated: 509-5p
6	PMID: 32498409	plasma exosomal miRNA	qRT-PCR	local ccRCC vs met	upregulated:<br>downregulated: 126-5p, 200c-3p
7	PMID: 28639257	plasma	qRT-PCR	ccRCC vs controls	upregulated: miR-26b#<br>downregulated: miR-451
8	PMID: 24379138	plasma	qRT-PCR	ccRCC vs controls	upregulated: 221 (met & ccRCC), 222 (ccRCC);<br>downregulated:
9	PMID: 25909813	plasma	qRT-PCR	RCC vs controls	upregulated: miR-7<br>downregulated:
10	PMID: 23916610	plasma	qRT-PCR	ccRCC vs controls	upregulated:<br>downregulated: miR-187
11	PMID: 28336290	serum	qRT-PCR	ccRCC vs control	upregulated: 1233<br>downregulated: 34a, 141
12	PMID: 32556891	serum	qRT-PCR	RCC vs controls	upregulated:<br>downregulated:
13	PMID: 26985942	serum	qRT-PCR	RCC vs controls	upregulated: 210<br>downregulated:
14	PMID: 27814278	serum	qRT-PCR	RCC vs controls	upregulated: 21, 106a<br>downregulated:
15	PMID: 29332334	serum	qRT-PCR	RCC vs controls	upregulated:<br>downregulated: miR429
16	PMID: 26091793	serum	qRT-PCR	RCC vs control	upregulated: 183<br>downregulated:
17	PMID: 29410711	serum	NGS validated by qRT-PCR	ccRCC vs benign	upregulated:<br>downregulated: 122-5p, 206
18	PMID: 32819588	serum	qRT-PCR	RCC	upregulated: miR-9-5p<br>downregulated:
19	PMID: 21984948	serum	validated in multicenter cohort	RCC	upregulated: 1233, 7-1*, 93, 106b*, 210, 320b, 1290<br>downregulated:
20	PMID: 28753793	serum exosomes	qRT-PCR	ccRCC vs control	upregulated: 1233, 210<br>downregulated:
21	PMID: 31661117	serum	qRT-PCR	ccRCC	upregulated: miR-885-5p, miR-592<br>downregulated: miR-141-3p, miR-508-3p
22	PMID: 23064048	serum	qRT-PCR	RCC vs control	upregulated: 210<br>downregulated:
23	PMID: 26426010	serum	qRT-PCR	RCC vs control	upregulated: 378, 210<br>downregulated:
24	PMID: 22542158	serum	qRT-PCR	ccRCC vs benign	upregulated: 378<br>downregulated:
25	PMID: 22440013	serum	qRT-PCR	RCC vs control	upregulated: 378<br>downregulated: 451
26	PMID: 34247370	serum exosomes	qRT-PCR	RCC vs controls	upregulated: 106a<br>downregulated:
27	PMID: 24212760	serum	qRT-PCR	RCC vs control	upregulated: 210<br>downregulated:
28	PMID: 26481440	serum		RCC vs control	upregulated:<br>downregulated:
29	PMID: 32823234	serum	qRT-PCR	RCC vs control	upregulated: 196a-5p<br>downregulated: 20b-5p, 30a-5p
30	PMID: 25556603	serum	qRT-PCR	early stage ccRCC vs controls	upregulated: 193a-3p, 362, 572<br>downregulated: 28-5p, 378
31	PMID: 33893078	serum EVs	arrays and qRT-PCR	Control vs localized RCC vs advanced RCC	upregulated: 4525, 4516, 4534, 614, 4448, 4497, 4710, 8059, 3960<br>downregulated:
32	PMID: 22369946	plasma	NGS	RCC vs controls	upregulated:<br>downregulated: 508-3p, 509-3p
33	PMID: 31488416	serum	ddPCR technology	NSCLC	upregulated:<br>downregulated:
34	PMID: 32703272	serum	ddPCR technology	ovarian cancer	upregulated:<br>downregulated:
35	PMID: 26036630	plasma/serum	ddPCR technology	breast cancer	upregulated:<br>downregulated:

Table S2. ddPCR Reaction Details.

In an effort to maximize the signal noise of each assay, three parameters were tested: assay type (miRCURY vs. TaqMan), the annealing and extension temperature, and the dilution of the input RT. The following conditions were found to be optimal and were used to test the samples in this study.

microRNA	assay information	annealing and extension	RT dilution
hsa-miR-16-5p	miRCURY YP0020570, Qiagen	54.7°C	1:2500
hsa-miR-93-5p	miRCURY YP00204715, Qiagen	54.7°C	1:50
hsa-miR-103a-3p	miRCURY YP00204063, Qiagen	55.9°C	1:50
hsa-miR-144-3p	miRCURY YP00204754, Qiagen	54.7°C	1:200
hsa-miR-210-3p	TaqMan 000512, Life Technologies	60°C	1:40
hsa-miR-221-3p	TaqMan 000524, Life Technologies	60°C	1:100
hsa-miR-222-3p	miRCURY YP00204551, Qiagen	54.7°C	1:10

LOQ Results

For ddPCR assays, serial dilution (1:2) of cDNA generated from plasma RNA was performed to determine the limit of quantification (LOQ) for the ddPCR assays. The LOQ was defined as the lowest concentration that could be detected with a CV ≤ 25% [36]. The LOQ was 0.40 copies/µl for miR-210-3p and 0.94 copies/µl for miR-222-3p (Figure S1).

Figure S1. Limit of quantification results. Serial dilution series for **(a)** miR-210-3p and **(b)** miR-222-3p.

(a)

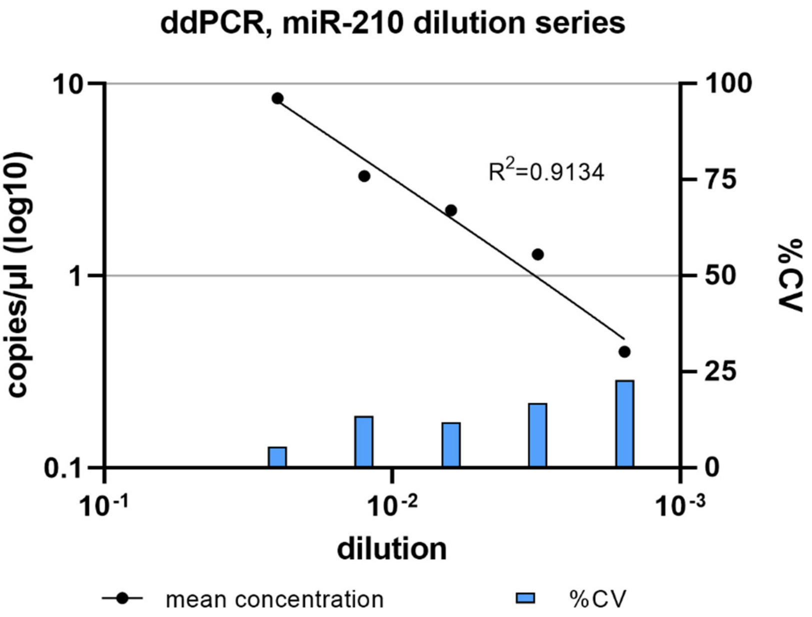

(b)

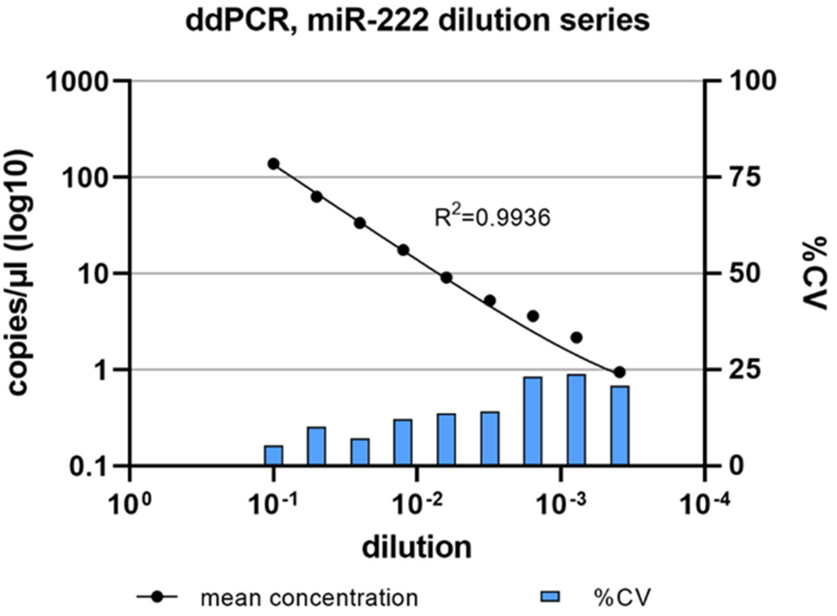

Reference

31. Muramatsu-Maekawa, Y.; Kawakami, K.; Fujita, Y.; Takai, M.; Kato, D.; Nakane, K.; Kato, T.; Tsuchiya, T.; Koie, T.; Miura, Y.; et al. Profiling of Serum Extracellular Vesicles Reveals miRNA-4525 as a Potential Biomarker for Advanced Renal Cell Carcinoma. *Cancer Genom. Proteom.* **2021**, *18*, 253–259, doi:10.21873/cgp.20256.
32. Zhai, Q.; Zhou, L.; Zhao, C.; Wan, J.; Yu, Z.; Guo, X.; Qin, J.; Chen, J.; Lu, R. Identification of miR-508-3p and miR-509-3p That Are Associated with Cell Invasion and Migration and Involved in the Apoptosis of Renal Cell Carcinoma. *Biochem. Biophys. Res. Commun.* **2012**, *419*, 621–626, doi:10.1016/j.bbrc.2012.02.060.
33. D'Antona, P.; Cattoni, M.; Dominioni, L.; Poli, A.; Moretti, F.; Cinquetti, R.; Gini, E.; Daffrè, E.; Noonan, D.M.; Imperatori, A.; et al. Serum miR-223: A Validated Biomarker for Detection of Early-Stage Non-Small Cell Lung Cancer. *Cancer Epidemiol. Biomark. Prev.* **2019**, *28*, 1926–1933, doi:10.1158/1055-9965.EPI-19-0626.
34. Cirillo, P.D.R.; Margiotti, K.; Mesoraca, A.; Giorlandino, C. Quantification of Circulating microRNAs by Droplet Digital PCR for Cancer Detection. *BMC Res. Notes* **2020**, *13*, 351, doi:10.1186/s13104-020-05190-3.
35. Ferracin, M.; Lupini, L.; Salamon, I.; Saccenti, E.; Zanzi, M.V.; Rocchi, A.; Da Ros, L.; Zagatti, B.; Musa, G.; Bassi, C.; et al. Absolute Quantification of Cell-Free microRNAs in Cancer Patients. *Oncotarget* **2015**, *6*, 14545–14555, doi:10.18632/oncotarget.3859.
36. Floren, C.; Wiedemann, I.; Brenig, B.; Schütz, E.; Beck, J. Species Identification and Quantification in Meat and Meat Products Using Droplet Digital PCR (ddPCR). *Food Chem* **2015**, *173*, 1054–1058, doi:10.1016/j.foodchem.2014.10.138.
